# Effectiveness of Interventions on Improving Balance in Children and Adolescents With Hearing Impairment: A Systematic Review

**DOI:** 10.3389/fphys.2022.876974

**Published:** 2022-05-16

**Authors:** Yan Zhou, Jing Qi

**Affiliations:** ^1^ Xingzhi College, Zhejiang Normal University, Jinhua, China; ^2^ College of Physical Education and Health Sciences, Zhejiang Normal University, Jinhua, China

**Keywords:** hearing impairment, balance, physical exercise, effectiveness, systematic review

## Abstract

Although children and adolescents with hearing impairment are at risks of falls from balance problems, reliable information on effects of interventions are scare. Therefore, the purpose of this review is to systematically summarize studies on the evidence of interventions to improve balance ability in children and adolescents with hearing impairment. A systematic literature search was conducted on five major electronic databases. Studies were included if: 1) interventions or trials focusing on improving balance in children and adolescents with hearing impairment; 2) research targeting children with hearing impairment (samples with a mean age below 18 years); 3) studies were published in English peer-reviewed journals due to language barriers and resource limitations; and 4) study designs were randomized controlled trial or quasi-experiment. A nine-item tool adapted from the Consolidated Standards of Reporting Trials Statement was used to assess the quality of the studies. Through the search strategy, 373 articles were identified, and 15 studies published between 1981 and 2021 met the inclusion criteria. Most of the studies reviewed were categorized as medium or low quality, and only three were identified as high quality. Exercise interventions were adopted in 80% of the included studies, whereas studies that employed music + vibration, motor, and game as the intervention modalities accounted for the remaining 20.0%. The results of this review showed that the included trials with exercise interventions had a positive influence on the balance among children and adolescents with hearing impairment (the post-intervention scores were significantly higher than the pre-intervention or the control group scores). In addition, the interventions with duration of 8–16 weeks were more effective than those with less than 8 weeks. However, due to most of the reviewed studies were of low methodological quality, the trials results analyzed by this systematic review should be interpreted with caution. Further investigations of high-quality studies are therefore needed to prove the effectiveness of interventions on improving balance performance in children and adolescents with hearing impairment.

**Systematic Review Registration:** [https://www.crd.york.ac.uk/PROSPERO/], PROSPERO [308803].

## Introduction

Balance is an essential prerequisite for most daily life activities in children (Melo et al., 2020). It is the complex ability to maintain, achieve, or restore the state of balance of the body while a child stands still, prepares to move in movement, or prepares to stop moving (Pollock et al., 2000). Balance requires the integration of several sensory, motor, and biomechanical inputs (Nashner et al., 1982). However, changes in some of these sensory systems (e.g., visual, somatosensory, and vestibular) can trigger disturbances in the body balance (Melo et al., 2020; Paillard, 2017). The capability of balance can be examined in static (the body remains motionless) or dynamic (the body can react to perturbations or is in movement) conditions, as well as in both conditions (Paillard, 2017). For children and adolescents, maintaining balance is an essential prerequisite to competently perform most activities of daily living and is important for the proficient performance of fundamental movement skills (Mickle et al., 2011). Studies have shown that the most significant transitions in motor development occur in the first decade of life with balance control usually established at 7–10 years old (Ferdjallah et al., 2002; Roncesvalles et al., 2001). Therefore, addressing characteristics in the balance ability of children and adolescents is important for interventions in many practical fields.

Hearing impairment refers to the complete and partial loss of the ability to hear (Mathers et al., 2000). According to the World Health Organization, over 5% of the world’s population are suffering from some form of hearing impairment and it is estimated that by 2050, over 700 million people (one in every 10 people) will have hearing impairment.^1^ Childhood hearing impairment is a significant public health problem, which is associated with long term academic and communicative difficulties (Davis et al., 2001) and other physical deficits (e.g., vestibular-related impairments) (Pajor and Jozefowicz-Korcxynska, 2008; Siegel et al., 1991). The previous studies have proved that children and adolescents with hearing impairment are at increased risks of motor and balance problems due to their balance and/or motor deficits related to damage to the vestibular system (Fellinger et al., 2015; McPhillips, 2015). Furthermore, studies have shown that hearing impairment is associated with an increased risk of all-cause mortality (Karpa et al., 2010), possibly *via* physical activity-related parameters, such as mobility function and balance (Lin and Ferrucci, 2012; Loprinzi, 2015).

Over the decades, a few intervention studies have been conducted to improvise balance in children and adolescents with hearing impairment on different outcome measures (Ebrahimi et al., 2017; Effgen, 1981; Rine et al., 2004). Only one review conducted by Fernandes et al. (2015) analyzed three trials on the management of vestibular and balance functions in children with hearing impairment and concluded that vestibular rehabilitation has a positive influence on balance outcomes. Numerous works examined effects of interventions on balance improvements in this population since 2015 and have not been summarized and reviewed. Therefore, the purpose of our review is to systematically summarize studies on the evidence of interventions to improve balance ability in children and adolescents with hearing impairment until 2021. Our findings can provide insights for clinical practice and future studies on balance improvement among children and adolescents with hearing impairment.

## Methods

This systematic review was conducted while adhering to the guidelines of the Preferred Reporting Items for Systematic Reviews and Meta-Analyses (PRISMA) Statement (Moher et al., 2009).

### Search Strategy

The researchers systematically searched studies with the following databases from inception to December 2021: PubMed, PsycINFO, Scopus, Education Resources Information Centre (ERIC), and Web of Science (WoS) Core Collection. The search strategy included four groups of keywords: 1) hearing impairment* OR hearing disability* OR deaf OR hard of hearing; 2) child* OR adolescent* OR teenager* OR youth* OR youngster* OR student* OR pupil*; 3) motor ability* OR motor skill* OR balance OR posture control OR postural stability; 4) intervention OR trial OR experiment. In addition, the snowballing technique was used to identify potential studies by scanning the references of all the included articles.

### Inclusion and Exclusion Criteria

Studies meeting the following criteria were included in this review: 1) interventions or trials focusing on improving balance in children and adolescents with hearing impairment; 2) research targeting children with hearing impairment (samples with a mean age below 18 years); 3) studies were published in English peer-reviewed journals due to language barriers and resource limitations; and 4) study designs were randomized controlled trial (RCT) or quasi-experiment (QE). Studies were excluded if they 1) were pertaining to other topics; 2) participants’ mean age was above 18; 3) were unpublished articles, comments, conference proceedings, reviews, and dissertations; and 4) failed to report the detailed outcomes of trials. Two authors independently screened the returned articles according to the inclusion and exclusion criteria. Any disagreements between authors were resolved through discussion.

### Quality Assessment

To determine the methodological quality of the included studies, the researchers used a nine-item tool adapted from the Consolidated Standards of Reporting Trials Statement (Schulz et al., 2010), which has been used to assess the methodological quality of previous systematic reviews in similar areas (Healy et al., 2021; Morgan et al., 2013) (Table 1). Two authors independently assessed all studies. Each item was scored as 1 (the assessed item was explicitly described and presented) or 0 (the assessed item was inadequately described or absent). If consensus could not be reached, then agreement was obtained through discussion between the authors. The score for each study was summed, and the median score for all included study scores was calculated. Articles were determined of high quality when they scored above the median score, medium quality when they scored equal to the median score, and low quality when they scored below the median score (Zeng et al., 2017).

**TABLE 1:** Risk of bias checklist.

Item	Description
1	Randomization (generation of allocation sequence, allocation concealment and implementation) clearly described and adequately completed
2	Valid measures (validation data were provided by the author)
3	Blinded outcome assessment (assessor blinding)
4	Participants analyzed in group they were originally allocated to, and participants not excluded from analyses because of noncompliance to treatment or because of missing data
5	Covariates accounted for in analyses (e.g., baseline score and other relevant covariates when appropriate such as age or sex)
6	Power calculation reported for main outcome
7	Presentation of baseline characteristics separately for treatment groups
8	Dropout was described, with a ≤20% dropout for studies with follow-up of≤6 months and ≤30% dropout for studies with follow-up>6 months
9	Summary results for each group + estimated effect size (difference between groups) + its precision (e.g., 95% CI)

### Data Extraction and Analysis

The first author completed the data extraction and then the second author verified the data. Discrepancies were resolved through a consensus discussion. The researchers extracted the following information: the first author, publication year, geographic location, and intervention detail.

Given the heterogeneity of the included studies, meta-analysis was not conducted. Instead, the intervention characteristics of each study were first summarized and analyzed and then recorded on a standardized form created by the authors. The effective rate of the interventions was calculated using the following equation: effective trials (the post-intervention scores were significantly higher than the pre-intervention or the control group scores)/(total number of trials). Similarly, data analysis was performed by the first author and then verified by the second author.

## Results

### Search Results

**FIGURE 1:**
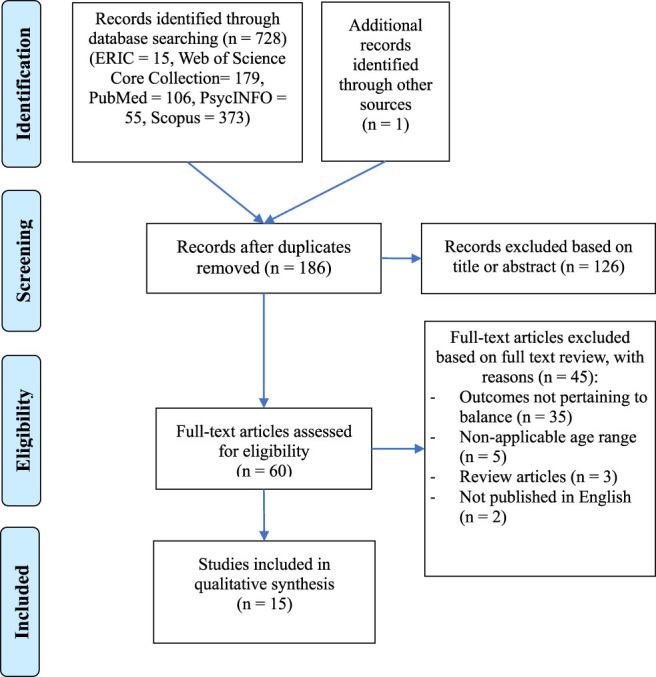
Study selection process.

The initial search identified 373 studies (ERIC, *n* = 15; WoS Core Collection, *n* = 179; PubMed, *n* = 106; PsycINFO, *n* = 55; Scopus, *n* = 373). Of these articles, 186 duplicates were removed. An additional article was identified from a citation search of relevant published literature reviews. After screening the titles and abstracts, 126 studies were excluded. The remaining full-text articles were read, and 45 others were excluded. Therefore, a total of 15 studies were included in this review. The PRISMA flowchart is shown in Figure 1.

### Methodological Quality

Table 2 outlines the results of the assessment of methodological quality. Overall, three out of the 15 included studies (20%) were categorized as high quality, and three (20%) and nine (60%) other studies were of medium and low quality, respectively. The weak components among the included studies were randomization, assessor blinding and result precision. Specifically, only 20% of the included studies described participant randomization, the blinded outcome assessment, and the outcome precision (e.g., 95% Confidence Interval).

**TABLE 2:** Results of study quality evaluation of included studies.

First Author (year)	Item 1	Item 2	Item 3	Item 4	Item 5	Item 6	Item 7	Item 8	Item 9	Score	Quality
Effgen (1981)	0	1	0	0	0	0	0	1	0	2	low
Fotiadou et al. (2002)	0	1	0	1	0	0	1	1	0	4	low
Rine et al. (2004)	0	1	1	1	0	0	1	1	0	5	medium
Borowiec (2011)	0	1	0	1	1	0	0	1	0	4	low
Rajendran et al. (2013)	1	1	0	1	0	1	1	1	1	7	high
Shah et al. (2013)	0	1	0	1	0	0	1	1	0	4	low
Majlesi et al. (2014)	0	1	0	1	0	1	1	1	0	5	medium
Karbunarova (2016)	0	1	0	0	0	0	1	1	0	3	low
Ebrahimi et al. (2017)	0	1	0	1	0	0	0	1	0	3	low
Vernadakis et al. (2018)	0	1	0	1	0	1	1	1	0	5	medium
Ilkim and Akyol (2018)	0	1	0	1	0	0	1	1	0	4	low
Demirel (2018)	0	1	0	1	0	0	0	1	0	3	low
Soori et al. (2019)	0	1	0	1	1	0	0	1	0	4	low
Zarei and Norasteh (2020)	1	1	1	0	1	1	1	0	1	7	high
Zarei et al. (2021)	1	1	1	0	1	1	1	0	1	7	high

### Study Characteristics

Table 3 presents the study characteristics. The included studies were published between 1981 and 2021; those published before 2000, 2001–2010, and 2011–2021 accounted for 6.7, 13.3, and 80.0%, respectively. These studies were from seven countries and most of which were from Asian countries, including Iran (5, 33.3%), India (2, 13.3%), and Turkey (2, 13.3%). Eleven studies (73.3%) were quasi-randomized trials, and the remaining (26.7%) were RCT designs. The samples included children (73.3%) and adolescents (26.7%), majority of which reported gender composition. Ten studies (66.7%) involved sample sizes between 20 and 40, whereas 20.0 and 13.3% had sample sizes less than 20 and more than 40, respectively. In addition, 66.7% of the included studies failed to report specific types of hearing impairment. Among the trials that reported the degree of hearing loss, 72.7% had moderate to severe hearing loss (with a hearing loss of 56–90 dB) and 27.3% had profound loss (hearing loss>90 dB).

**TABLE 3:** Characteristics of included studies.

First Author (year, country)	Design/Sample Characteristic	Modality	Intervention	Instruments (outcomes)	Method of Statistics	Effect
Effgen (1981, United States)	QE *n* = 49 (29 male)	Motor	10 days. ① 15 min/day, Standing activities of static balance; ② Normal classroom activity (free play).	Force platform (Static balance and stability)	independent *t*-test	① > ② (Time of standing on one leg)
	M age: 9.1 hearing impairment type: NR					① ≈ ② (Sway degree)
	Hearing loss >75dB					
Fotiadou (2002, Greece)	QE *n* = 29 (15 male)	Exercise	16 weeks. ① 3 × 40 min/week, Rhythmic gymnastics activities; ② 3 × 40 min/week, Physical education activities.	Stabilometer (Dynamic balance)	independent *t*-test; paired *t*-test	① > ②
	M age: 7.9/8.0 hearing impairment type: SNHL					
	Hearing loss >70dB					
Rine (2004, United States)	RCT *n* = 21 (9 male)	Exercise	12 weeks. ① 3 × 30 min/week, Exercise intervention focused on substitution, including eye hand coordination, general coordination activities, visual motor training, and balance training; ② 3 × 30 min/week, Language development training.	PSCT (Stability)	independent *t*-test; Wilcoxon signed rank tests	① > ②
	M age: 5.6/5.7 hearing impairment type: SNHL + VI					
	Hearing loss: moderate to profound					
Borowiec (2011, Poland)	QE *n* = 25 (12 male)	Music + vibration	16 weeks. ① 1 × 45 min/week, Physical exercise performed to the music with enhanced high frequencies and vibration devices; ② 1 × 45 min/week, Traditional dancing classes using every day hearing aids.	Balancing Backward	Mann–Whitney *U*-test	① > ②
	age range: 10–13			Test (Dynamic balance)		
	hearing impairment type: NR					
	Hearing loss: NR					
Rajendran (2013, India)	RCT *n* = 21 (14 male)	Exercise	6 weeks. ① 3 × 45 min/week, Vestibular-specific neuromuscular training, including training of balance retraining, Eye-hand coordination and FMS; ② Regular school activities.	One Leg Standing Balance Test/Postural sway meter and Pediatric Reach Test (Static balance and stability)	Mann–Whitney *U*-test	① > ②
	M age: 7.5/8.1 hearing impairment type: NR					
	Hearing loss >90dB					
Shah (2013, India)	QE (Pre-post test) *n* = 10 (6 male)	Exercise	12 weeks. 3 × 45 min/week, exercise sessions including eye-hand and general coordination exercises, visual motor training, balance training.	Pediatric Balance Scale (Overall balance)	paired *t*-test	① > ②
	M age: 7.6 hearing impairment type: SNHL					
	Hearing loss: NR					
Majlesi (2014, Iran)	QE *n* = 20 (20 male)	Exercise	4 weeks. ① 3 × 45 min/week, Exercise balance program based on proprioception training; ② NR.	Force platform (Static balance and stability)	independent *t*-test; repeated-measures ANOVA	① > ②
	M age: 10.4/11.3 hearing impairment type: NR					
	Hearing loss >75dB					
Karbunarova (2016, Ukraine)	QE *n* = 20 (16 male)	Exercise	10 weeks. ① 2 times/week, Swimming classes; ② 2 times/week, Soccer, volleyball and basketball classes.	Romberg and Bondarevsky’s difficult test/Walking on balance beam test (Static and dynamic balance)	independent *t*-test; paired *t*-test	① > ②
	age range: 6–10					
	hearing impairment type: NR					
	Hearing loss: NR					
Ebrahimi (2017, Iran)	QE *n* = 24 (18 male)	Exercise	8 weeks. ① 3 × 45 min/week, Progressive exercise program including adaption, eye-head coordination and substitution exercises; ② NR.	PSOT (Static balance and stability)	independent *t*-test; paired *t*-test	① > ②
	M age: 9.8/10.1 hearing impairment type: SNHL + VI					
	Hearing loss>90dB					
Demirel (2018, Turkey)	QE *n* = 18 (12 male)	Exercise	10 weeks. ① 2 × 50–75 min/week, Special movement training program; ② NR.	Gross motor development tests (Dynamic balance)	Mann–Whitney *U*-test	① > ②
	M age: 7.4 hearing impairment type: NR					
	Hearing loss: NR					
Vernadakis (2018, Greece)	QE *n* = 20 (10 male)	Game	8 weeks. ① 2 × 15 min/week, Interactive games Wii-Fit Plus of the Nintendo Wii console; ② Traditional adapted physical education balance training program.	Flamingo Balance Test (Static balance)	independent *t*-test;	① ≈ ②
	M age: 18.3 hearing impairment type: SNHL				repeated-measures ANOVA	
	Hearing loss >70dB					
Ilkim (2018, Turkey)	QE *n* = 60 (NR)	Exercise	14 weeks. ① 3 times/week, Athletic exercises; ② 3 times/week, Gymnastic exercises.	Flamingo Balance Test (Static balance)	independent *t*-test; paired *t*-test	① > ②
	M age: 12.4/12.8 hearing impairment type: NR					
	Hearing loss: 90–110 dB					
Soori (2019, Iran)	QE *n* = 20 (20 female)	Exercise	8 weeks. ① 3 × 60 min/week, Perceptual-motor training (such as balance training, running between obstacles); ② Daily routine works.	Stork balance test/Y Balance Test (Static and dynamic balance)	independent *t*-test; paired *t*-test	① > ②
	M age: 9.35 hearing impairment type: NR					
	Hearing loss >61dB					
Zarei (2020, Iran)	RCT *n* = 20 (20 male)	Exercise	8 weeks. ① 3 × 60 min/week, Proprioception training without visual input; ② Daily activities.	Single-limb standing test (Static balance)	repeated-measures ANOVA	① > ②
	M age: 16.4/16.9 hearing impairment type: NR					
	Hearing loss >75dB					
Zarei (2021, Iran)	RCT *n* = 19 (19 female)	Exercise	8 weeks. ① 3 × 60 min/week, Pilates training program; ② Daily activities.	Balance Errors Test/Y Balance Test (Static and dynamic balance)	repeated-measures ANOVA	① > ②
	M age: 16.7 hearing impairment type: NR					
	Hearing loss >75dB					

Note. QE, quasi-experiment; RCT, randomized controlled trial; M age, mean age; dB, decibel; SNHL, sensorineural hearing loss; ①, intervention group; ②, control group; In the Pre-post test, ① post-test evaluation,② pre-test evaluation; VI, vestibular impairment; PSCT, Posturography sensory conditions testing; PSOT, posturography sensory organization testing; FMS, fundamental motor skill; NR, not reported.

### Intervention Characteristics

Table 4 presents the intervention characteristics.

**TABLE 4:** Summary of intervention characteristics of included studies.

Description	Category	n (%)	Effective Rate (%)	Description	Category	n (%)	Effective Rate (%)
Year of publication	<2000	1 (6.7%)	50.0%	Duration	<8 weeks	3 (20.0%)	75.0%
	2001–2010	2 (13.3%)	100.0%		8–16 weeks	12 (80.0%)	91.7%
	2011–2021	12 (80.0%)	91.7%		1 time/day	1 (6.7%)	50%
Country	Iran	5 (33.3%)	100.0%	Frequency	1 time/week	1 (6.7%)	100%
	India	2 (13.3%)	100.0%		2 times/week	3 (20.0%)	66.7%
	Turkey	2 (13.3%)	100.0%		3 times/week	10 (66.7%)	100%
	United States	2 (13.3%)	66.7%	Session (min)	≤15	2 (13.3%)	33.3%
	Greece	2 (13.3%)	50.0%		30–45	7 (46.7%)	100%
	Poland	1 (6.7%)	100.0%		>45	4 (26.7%)	100%
	Ukraine	1 (6.7%)	100.0%		NR	2 (13.3%)	100%
Design	RCT	4 (26.7%)	100.0%	Instruments	One-Leg Standing Balance Test	5 (26.3%)	80%
	QE	11 (73.3%)	83.3%		Y Balance Test	2 (10.5%)	100%
Sample size	<20	3 (20.0%)	100.0%	—	Pediatric Balance Scale	1 (5.3%)	100.0%
	20–40	10 (66.7%)	90.0%		Force Platform	2 (10.5%)	66.7%
	>40	2 (13.3%)	66.7%		Other instrument	9 (47.4%)	100.0%
Mean age (year)	6–12	11 (73.3%)	91.7%	Outcomes	Dynamic Balance	6 (27.3%)	100%
	13–18	4 (26.7%)	75.0%		Static Balance	10 (45.5%)	90%
hearing impairment type	SNHL	5 (33.3%)	80%	—	Overall Balance	1 (4.5%)	100%
	NR	10 (66.7%)	90.9		Stability	5 (22.7%)	80%
Hearing loss (dB)	56–90	8 (53.3%)	77.8	—	—	—	—
	>90	3 (20.0%)	100.0		—	—	—
	NR	4 (26.7%)	100.0		—	—	—
Modality	Exercise training	12 (80.0%)	100.0	—	—	—	—
	Music + vibration	1 (6.7%)	100.0		—	—	—
	Motor intervention	1 (6.7%)	50.0		—	—	—
	Game intervention	1 (6.7%)	0		—	—	—

Note. NR, not reported.

#### Intervention Modality

Exercise interventions were adopted in most studies (12, 80.0%), whereas studies that employed music + vibration, motor, and game as the intervention modalities accounted for the remaining 20.0%. Exercise intervention mainly used a series of structured course or training, such as vestibular-specific neuromuscular training (Rajendran et al., 2013), exercise balance program based on proprioception (Majlesi et al., 2014) and so on. High-frequency music and vibration equipment were used in music + vibration intervention. In motor intervention, the 15-min of standing movements in different positions were chosen (Effgen, 1981). While Nintendo Wii-Fit Plus interactive games were selected for game intervention (Vernadakis et al., 2018).

#### Duration and Frequency

The total duration of interventions ranged from 10 days to 16 weeks, 80% of which opted for a duration of 8–16 weeks. In terms of intervention frequency, 66.7% of the included studies performed their experiments three times a week, whereas 20.0 and 6.7% conducted their experiments twice and once a week, respectively. Sessions of 30–45 min per time were selected by 46.7% of the trials, whereas sessions of more than 45 min and less than 15 min per time accounted for 26.7 and 13.3%, respectively. However, 13.3% of the trials failed to report the duration of each session.

#### Outcome Measure

A total of 10 studies evaluated the static balance, among which the most common assessment was one-leg standing test, such as One-leg Standing Balance Test, Stork Balance Test, Flamingo Balance Test, and Single-limb Standing Test. Six studies tested dynamic balance, two of which used Y Balance Test. Five other studies tested subjects’ postural stability, and another one measured overall balance using the Pediatric Balance Scale.

#### Intervention Effect

Overall, 13 out of 15 trials (86.7%) showed a positive effect on improving balance among children and adolescents with hearing impairment (the post-intervention scores were significantly higher than the pre-intervention or the control group scores). Interventions with a total duration of 8–16 weeks were more effective than those with less than 8 weeks (91.7 *vs*. 75.0%). And trials in children with hearing impairment were more effective than adolescents with hearing impairment (91.7 *vs*. 75.0%). Besides, among 10 studies that measured static balance, the effective rate was 90%, whereas the efficiency of six studies that assessed dynamic balance was 100%. Five studies that evaluated postural stability had an effective rate of 80%, whereas the effective rate of one study that measured overall balance was 100%.

#### Method of Statistics

Nearly half of the studies (46.7%) conducted independent t-test or paired t-test, and 20.0% of which performed U-test. Repeated-measures ANOVA and its combination with t-test accounted for 13.3% of the total, whereas t-test combined with Wilcoxon signed rank tests accounted for 6.7% of the studies.

## Discussion

For this review, we identified 15 studies that used the intervention program to improve the balance of children and adolescents with hearing impairment. The results of methodological quality assessment showed that most of the studies reviewed were categorized as medium or low quality, and only three were identified as high quality. Most research used QE designs, being one of the reasons that reduce the methodology quality. Even in the RCT designs reviewed, the random assignment, allocation concealment, and assessor blinding procedures were not fully described. RCT designs pose methodical, practical, and ethical challenges to researchers (Hein & Weeland, 2019), especially in populations with social or cognitive impairments, such as children and adolescents with hearing impairment (Mulhall et al., 2018). Nevertheless, randomized trials and their systematic reviews can provide the most reliable evidence about the effects of healthcare interventions (Higgins et al., 2011).

One area that must be pointed in the reviewed trials is the relatively small sample sizes. Most studies (86.7%) had sample sizes less than 40, and none of the authors specified if they used calculations to establish such sample sizes. Theoretically, sample size depends on three aspects: the main measurement variable, the variance in the primary variable, and the acceptable error. Bartlett et al. (2001) described the procedures for determining the appropriate sample sizes for different types of variables, which may provide some methodological references for researchers. Moreover, budget and experiment feasibility are other aspects that must be considered in determining sample sizes.

Another shortcoming of some trials analyzed in this review is not controlling the types of hearing impairment. According to the American Speech–Language–Hearing Association, hearing loss has three basic types: conductive hearing loss, sensorineural hearing loss (SNHL), and mixed hearing loss (Fernandes et al., 2015). However, only 33.3% of the included studies reported a type of hearing impairment, that is, SNHL. The remaining works failed to report a specific type. SNHL is the most common type of permanent hearing loss, which is caused by functional problems in the cochlea or the auditory pathway to the brain (McPhillips, 2015). It has been reported that children with SNHL as opposed to conductive hearing loss, have progressive developmental delay, and is related to concomitant damage to vestibular structure (Rine et al., 2004). Thus if the trials included children with and without SNHL and the results were not reported separately or did not control the vestibular dysfunction among the subjects with SNHL, this may underestimate the effect size of the interventions. Controlling the type of hearing impairment in future trials may guide rehabilitation strategies specifically, help easily obtain comparison results, and synthesize pieces of trial evidence.

In terms of the instruments for the evaluation of the outcomes, future studies should fully consider the applicability of measurement tools to children and adolescents with hearing impairment. The use of unvalidated instruments would reduce the reliability of the evidence (Melo et al., 2020). Romberg test, for example, which was proposed for the evaluation of the elderly balance, may not be suitable for balance assessment in children and adolescents with hearing impairment.

The results from this review showed that the included trials with exercise interventions had a positive influence on the balance among children and adolescents with hearing impairment (the post-intervention scores were significantly higher than the pre-intervention or the control group scores). Given that most studies employed structured exercise training with an emphasis on balance and coordination that present wide displacements of the gravity center, the experimental group therefore may obtain further practice on the balance ability (Melo et al., 2020). Moreover, some exercise interventions focused on improving substitution (Ebrahimi et al., 2017; Rajendran et al., 2013; Rine et al., 2004; Shah et al., 2013). The neuromuscular control on balance was improved as a result of enhancing substitution through the development of visual and somatosensory awareness and incorporation of fundamental motor skills. Even so, we still need to look further into the neurological basis of balance and to design programs to improve the psychomotor integration of all factors that affecting the balance ability of children and adolescents with hearing impairment.

Another important finding of this review showed that the balance interventions were more effective in participants with an average age of 6–12 years than those with an average age of 13–18 years. This finding expanded, as well as affirmed, previous studies, that the most critical period in motor development occur in the first decade of life with balance control usually established at 7–10 years old (Ferdjallah et al., 2002; Roncesvalles et al., 2001). However, due to the processes that are responsible for resolving multi modal sensory conflict of postural stability are not fully developed before the age of 7 years old (Shumway-Cook and Woollacott, 1985), the future studies should categorize age groups when include children under 7 years old, in order to observe the results of the interventions in children with postural stability mature or in development (Melo et al., 2019).

Regarding the duration of training, interventions with duration of 8–16 weeks were more effective than those with less than 8 weeks. Short-duration programs may not suffice to support the physical and cognitive integration of new skills to achieve the long-term modification of balance ability (Maiano et al., 2019). Planning an exercise program of appropriate duration to promote full realization of each child’s balance potential therefore may be more helpful. Meanwhile, it should also be noted that the experimenter or assistant can maintain effective non-verbal communication with the subjects.

Studies that employed music + vibration, motor, and game as the intervention modalities had an effective rate of 100, 50, and 0%, respectively. Considering that each of these three modalities was adopted by only one study, drawing a conclusion on the effectiveness of these interventions on enhancing balance among children and adolescents with hearing impairment is difficult. Additional studies are needed to confirm the results of interventions adopting these modalities.

## Conclusion

Based on our findings, we conclude that the exercise interventions were effective on improving balance in children and adolescents with hearing impairment. In addition, the interventions with duration of 8–16 weeks were more effective than those with less than 8 weeks. However, most of the reviewed studies were of low methodological quality; thus, the trial results analyzed in this systematic review should be interpreted with caution. Further investigations of high-quality studies are therefore needed to prove the effectiveness of interventions on improving balance performance in children and adolescents with hearing impairment.

## Limitations

Two limitations inherent within the current review should be noted. First, although we conducted an extensive literature search on five major databases to identify potential studies, a few published studies were possibly missed because our search was limited to English journal articles. Certain studies, which could have added relevant information to the field, might have been discarded. Second, due to the heterogeneity of reviewed studies, such as participant characteristics and outcome measures, a meta-analysis could not be conducted.

## Data Availability

The original contributions presented in the study are included in the article/Supplementary Material, further inquiries can be directed to the corresponding author.

## Footnotes

1
https://www.who.int/news-room/fact-sheets/detail/deafness-and-hearing-loss.

## References

[B1] Bartlett J. E. Kotrlik J. W. Higgins C. C. (2001). Organizational Research: Determining Appropriate Sample Size in Survey Research. Inf. Technol. Learn. Perform. J. 19, 43–50.

[B2] Borowiec J. (2011). Possibilities of Application of Music and Vibrations for Improving Motor Coordination Abilities in Children with Impaired Hearing-Pilot Experiment Report. Fizjoterapia 19, 28–42. 10.2478/v10109-011-0009-3

[B4] Davis A. Bamford J. Stevens J. (2001). Performance of Neonatal and Infant Hearing Screens: Sensitivity and Specificity. Br. J. Audiology 35, 3–15. 10.1080/03005364.2001.11742727 11314908

[B5] Demirel N. (2018). The Impact of Therapeutic Recreational Gymnastic Exercise on Basic Motor Skills of Hearing-Impaired Children Aged between 6 and 9 Years. Jets 6, 147–151. 10.11114/jets.v6i3.3048

[B6] Ebrahimi A. A. Jamshidi A. A. Movallali G. Rahgozar M. Haghgoo H. A. (2017). The Effect of Vestibular Rehabilitation Therapy Program on Sensory Organization of Deaf Children with Bilateral Vestibular Dysfunction. Acta. Med. Iran. 55, 683–689. 29307157

[B7] Effgen S. K. (1981). Effect of an Exercise Program on the Static Balance of Deaf Children. Phys. Ther. 61, 873–877. 10.1093/ptj/61.6.873 7243886

[B8] Fellinger M. J. Holzinger D. Aigner M. Beitel C. Fellinger J. (2015). Motor Performance and Correlates of Mental Health in Children Who Are Deaf or Hard of Hearing. Dev. Med. Child. Neurol. 57, 942–947. 10.1111/dmcn.12814 26062643

[B9] Ferdjallah M. Harris G. F. Smith P. Wertsch J. J. (2002). Analysis of Postural Control Synergies during Quiet Standing in Healthy Children and Children with Cerebral Palsy. Clin. Biomech. 17, 203–210. 10.1016/S0268-0033(01)00121-8 11937258

[B10] Fernandes R. Hariprasad S. Kumar V. K. (2015). Physical Therapy Management for Balance Deficits in Children with Hearing Impairments: A Systematic Review. J. Paediatr. Child. Health 51, 753–758. 10.1111/jpc.12867 25808937

[B11] Fotiadou E. Giagazoglou P. Kokaridas D. Angelopoulou N. Tsimaras V. Tsorbatzoudis C. (2002). Effect of Rhythmic Gymnastics on the Dynamic Balance of Children with Deafness. Eur. J. Special Needs Educ. 17, 301–309. 10.1080/08856250210162211

[B12] Healy S. Obrusnikova I. Getchell N. (2021). Fundamental Motor Skill Interventions in Children with Autism Spectrum Disorder: A Systematic Review of the Literature Including a Methodological Quality Assessment. Res. Autism Spectr. Disord. 81, 101717. 10.1016/j.rasd.2020.101717

[B13] Hein S. Weeland J. (2019). Introduction to the Special Issue. Randomized Controlled Trials (RCTs) in Clinical and Community Settings: Challenges, Alternatives, and Supplementary Designs. New Dir. Child Adolesc. Dev. 2019, 7–15. 10.1002/cad.20312 31509328

[B14] Higgins J. P. T. Altman D. G. Gotzsche P. C. Juni P. Moher D. Oxman A. D. (2011). The Cochrane Collaboration's Tool for Assessing Risk of Bias in Randomised Trials. Bmj 343, d5928. 10.1136/bmj.d5928 22008217 PMC3196245

[B15] Ilkım M. Akyol B. (2018). The Comparison of Some Motoric Characteristics of Hearing Impaired Individuals Sports Athletic and Gymnastic. ujer 6, 2148–2152. 10.13189/ujer.2018.061012

[B16] Karbunarova J. (2016). Influence Author Methodic Teaching Swimming on Coordination Quality of Children 6-10 Years Old with Hearing Disabilities. Slobozhanskyi Her. Sci. Sport 3, 35–38. 10.15391/snsv.2016-3.012

[B17] Karpa M. J. Gopinath B. Beath K. Rochtchina E. Cumming R. G. Wang J. J. (2010). Associations between Hearing Impairment and Mortality Risk in Older Persons: The Blue Mountains Hearing Study. Ann. Epidemiol. 20, 452–459. 10.1016/j.annepidem.2010.03.011 20470972

[B18] Lin F. R. Ferrucci L. (2012). Hearing Loss and Falls Among Older Adults in the United States. Arch. Intern. Med. 172, 369–371. 10.1001/archinternmed.2011.728 22371929 PMC3518403

[B19] Loprinzi P. D. (2015). Accelerometer-determined Physical Activity and Mortality in a National Prospective Cohort Study: Considerations by Hearing Sensitivity. Am. J. Audiol. 24, 569–572. 10.1044/2015_AJA-15-0044 26650870

[B20] Maïano C. Hue O. Morin A. J. S. Lepage G. Tracey D. Moullec G. (2019). Exercise Interventions to Improve Balance for Young People with Intellectual Disabilities: a Systematic Review and Meta‐analysis. Dev. Med. Child. Neurol. 61, 406–418. 10.1111/dmcn.14023 30230530

[B21] Majlesi M. Farahpour N. Azadian E. Amini M. (2014). The Effect of Interventional Proprioceptive Training on Static Balance and Gait in Deaf Children. Res. Dev. Disabil. 35, 3562–3567. 10.1016/j.ridd.2014.09.001 25241115

[B22] Mathers C. Smith A. Concha M. (2000). Global Burden of Hearing Loss in the Year 2000. Glob. Burd. Dis. 18, 1–30.

[B23] McPhillips M. (2015). Motor Difficulties and Mental Health in Children Who Are Deaf. Dev. Med. Child. Neurol. 57, 893–894. 10.1111/dmcn.12831 26096193

[B24] Melo R. S. Lemos A. Paiva G. S. Ithamar L. Lima M. C. Eickmann S. H. (2019). Vestibular Rehabilitation Exercises Programs to Improve the Postural Control, Balance and Gait of Children with Sensorineural Hearing Loss: A Systematic Review. Int. J. Pediatr. Otorhinolaryngology 127, 109650. 10.1016/j.ijporl.2019.109650 31466025

[B25] Melo R. S. Tavares-Netto A. R. Delgado A. Wiesiolek C. C. Ferraz K. M. Belian R. B. (2020). Does the Practice of Sports or Recreational Activities Improve the Balance and Gait of Children and Adolescents with Sensorineural Hearing Loss? A Systematic Review. Gait Posture 77, 144–155. 10.1016/j.gaitpost.2020.02.001 32036319

[B26] Mickle K. J. Munro B. J. Steele J. R. (2011). Gender and Age Affect Balance Performance in Primary School-Aged Children. J. Sci. Med. Sport 14, 243–248. 10.1016/j.jsams.2010.11.002 21276751

[B27] Moher D. Liberati A. Tetzlaff J. Altman D. G. (2009). Preferred Reporting Items for Systematic Reviews and Meta-Analyses: The PRISMA Statement. J. Clin. Epidemiol. 62, 1006–1012. 10.1016/j.jclinepi.2009.06.005 19631508

[B28] Morgan P. J. Barnett L. M. Cliff D. P. Okely A. D. Scott H. A. Cohen K. E. (2013). Fundamental Movement Skill Interventions in Youth: A Systematic Review and Meta-Analysis. Pediatrics 132, e1361–e1383. 10.1542/peds.2013-1167 24167179

[B29] Mulhall P. Taggart L. Coates V. McAloon T. Hassiotis A. (2018). A Systematic Review of the Methodological and Practical Challenges of Undertaking Randomised-Controlled Trials with Cognitive Disability Populations. Soc. Sci. Med. 200, 114–128. 10.1016/j.socscimed.2018.01.032 29421458

[B30] Nashner L. Black F. Wall C. (1982). Adaptation to Altered Support and Visual Conditions during Stance: Patients with Vestibular Deficits. J. Neurosci. 2, 536–544. 10.1523/jneurosci.02-05-00536.1982 6978930 PMC6564270

[B31] Paillard T. (2017). Plasticity of the Postural Function to Sport And/or Motor Experience. Neurosci. Biobehav. Rev. 72, 129–152. 10.1016/j.neubiorev.2016.11.015 27894829

[B32] Pajor A. Jozefowicz-Korczynska M. (2008). Prognostic Factors for Vestibular Impairment in Sensorineural Hearing Loss. Eur. Arch. Otorhinolaryngol. 265, 403–407. 10.1007/s00405-007-0473-z 17926054

[B33] Pollock A. S. Durward B. R. Rowe P. J. Paul J. P. (2000). What Is Balance? Clin. Rehabil. 14, 402–406. 10.1191/0269215500cr342oa 10945424

[B34] Rajendran V. Roy F. G. Jeevanantham D. (2013). A Preliminary Randomized Controlled Study on the Effectiveness of Vestibular-specific Neuromuscular Training in Children with Hearing Impairment. Clin. Rehabil. 27, 459–467. 10.1177/0269215512462909 23076993

[B35] Rine R. M. Braswell J. Fisher D. Joyce K. Kalar K. Shaffer M. (2004). Improvement of Motor Development and Postural Control Following Intervention in Children with Sensorineural Hearing Loss and Vestibular Impairment. Int. J. Pediatr. Otorhinolaryngology 68, 1141–1148. 10.1016/j.ijporl.2004.04.007 15302144

[B36] Roncesvalles M. N. C. Woollacott M. H. Jensen J. L. (2001). Development of Lower Extremity Kinetics for Balance Control in Infants and Young Children. J. Mot. Behav. 33, 180–192. 10.1080/00222890109603149 11404213

[B37] Schulz K. F. Altman D. G. Moher D. (2010). CONSORT 2010 Statement: Updated Guidelines for Reporting Parallel Group Randomised Trials. J. Clin. Epidemiol. 63, 834–840. 10.1016/j.jclinepi.2010.02.005 20346629

[B38] Shah J. Rao K. Malawade M. Khatri S. (2013). Effect of Motor Control Program in Improving Gross Motor Function and Postural Control in Children with Sensorineural Hearing Loss-A Pilot Study. Pediat. Ther. 03, 1–4. 10.4172/2161-0665.1000141

[B39] Shumway-Cook A. Woollacott M. H. (1985). The Growth of Stability. J. Mot. Behav. 17, 131–147. 10.1080/00222895.1985.10735341 15140688

[B40] Siegel J. C. Marchetti M. Tecklin J. S. (1991). Age-related Balance Changes in Hearing-Impaired Children. Phys. Ther. 71, 183–189. 10.1093/ptj/71.3.183 2000434

[B41] Soori Z. Heyrani A. Rafie F. (2019). Exercise Effects on Motor Skills in Hearing-Impaired Children. Sport Sci. Health 15, 635–639. 10.1007/s11332-019-00564-y

[B42] Vernadakis N. Papastergiou M. Giannousi M. Panagiotis A. (2018). The Effect of an Exergame-Based Intervention on Balance Ability on Dear Adolescents. Sport Sci. 11, 36–41.

[B43] Zarei H. Norasteh A. A. Rahmanpournashrudkoli A. Hajihoseini E. (2020). The Effects of Pilates Training on Static and Dynamic Balance of Female Deaf Students: A Randomized Controlled Trial. J. Bodyw. Mov. Ther. 24, 63–69. 10.1016/j.jbmt.2020.05.003 33218566

[B44] Zarei H. Norasteh A. A. (2020). The Effect of 8 Weeks Proprioception Training without Visual Input on Single-Limb Standing Balance Time in Deaf Students: A Randomized Controlled Trial. J. Bodyw. Mov. Ther. 24, 63–68. 10.1016/j.jbmt.2019.09.002 32507154

[B45] Zeng N. Ayyub M. Sun H. Wen X. Xiang P. Gao Z. (20172017). Effects of Physical Activity on Motor Skills and Cognitive Development in Early Childhood: A Systematic Review. BioMed Res. Int. 2017, 1–13. 10.1155/2017/2760716 PMC5745693 29387718

